# Frequency of Intestinal Parasites among Zoo Animal by Morphometric Criteria and First Report of the *Bivitellobilharzia nairi* from Elephant (*Elephasmaximus maximus*) in Iran

**Published:** 2018

**Authors:** Aliyar MIRZAPOUR, Hamed KIANI, Iraj MOBEDI, Adel SPOTIN, Seyyed Javad SEYYED TABAEI, Mohsen RAHIMI

**Affiliations:** 1. Dept. of Medical Parasitology and Mycology, School of Medicine, Shahid Beheshti University of Medical Sciences, Tehran, Iran; 2. Iranian Organization of Veterinary, Hamedan, Iran; 3. Dept. of Medical Parasitology and Mycology, School of Public Health, Tehran University of Medical Sciences, Tehran, Iran; 4. Immunology Research Center, Tabriz University of Medical Sciences, Tabriz, Iran; 5. Dept. of Parasitology and Mycology, School of Medicine, Baqiyatallah University of Medical Sciences, Tehran, Iran

**Keywords:** Intestinal parasites, Morphometric criteria, Zoo animal, *Bivitellobilharzia nairi*, Iran

## Abstract

**Background:**

Intestinal parasitic infections are major causative agents of wildlife health complications among different parts of the world. This study aimed to investigate the gastro-intestinal parasites in feces of the zoo animals based on parasitological and morphometric criteria.

**Methods:**

One hundred fresh fecal samples were collected from 35 species of animal lived in Eram park zoo, Tehran, Central Iran during Oct 2015 to Jun 2015. All collected samples were examined by microscopic observation following direct wet mount preparation (normal saline and Lugol’s iodine), formalin-ether concentration, and permanent staining. The morphometric aspects of the recovered eggs were surveyed with the aid of Camera Lucida (×400).

**Results:**

65.7% (23/35) of zoo animal species were infected with intestinal parasites. The superfamily Trichostrongyloidea (6/16) and *Strongylus* sp. (16/4) were the most prevalent helminthic infections, while *Blastocystis* sp. (6/14), *Entamoeba* cyst (3/14) and *Eimeria* sp. (3/14) were the common protozoan parasites. For the first time, *Bivitellobilharzia nairi* egg was identified an elephant at Iran. Intestinal parasitic infections were apparently circulating among animals of the Eram park zoo.

**Conclusion:**

Identified parasitic infections can consider as a threatening source to visitors and workers’ health that have contact with animals or their feces. Therefore, the effectual preventive strategies should be addressed to determine the risk factors, mechanisms of cross-transmission of parasite, the importance of applying the hygienic practices and well adjusting deworming programs for the animals, zoo workers and visitors.

## Introduction

Animals (zoo animal, wild animal, and domestics) have important role as host and reservoir of many zoonosis parasites. Zoological parks exhibit wild animals (mammals, birds, reptiles, amphibians, fish, etc.,) for aesthetic, educational, and conservation purposes (1). However, the intestinal parasitic diseases are one of the major health concerns causing illness and even mortality among zoo animals while in captivity, the effects of which range from subclinical to death (2). Parasitic diseases play a principal role in animal health ranging from negative impacts on the host population size to the evolution of host behaviors. Parasitic infections constitute one of the main challenges in wild animals in captivity (3–5). Insufficient data about parasitic contaminations among zoo animals in Iran is a major limiting issue in zoological gardens. Identification of parasitic infections is important to study the prevalence, geographical distribution, taxonomic status and biology of parasites (6). There is no hesitation that a regular and consistent program of gastrointestinal parasite investigation and measures of control based on accurate diagnosis, effective treatment and suitable prophylaxis would surely assist in reversing the condition of ill health in zoo animals. By trying to establish a profile of intestinal parasites in the zoo animals, valuable data will be gained for the development of public health and preventive remedy. Evaluation of intestinal parasites in zoo animals and different geographic regions has medical and veterinary importance to prevent transmission of intestinal parasitic diseases to human and domestic animals. Many studies have been documented the intestinal parasites of the zoo animals in different populations of the world (1,2,5–8).

Schistosomatidae families are helminthes with worldwide distribution, almost lived in all temperate condition and tropical regions. *Schistosoma nairi* (*Bivitellobilharzia nairi*) is a blood fluke (the blood vessels of the liver parenchyma) of Asian and African elephants (namely *B. loxodontae*) and usually remains sub-clinical. The first report of *B. nairi* reported in 1955 and named it as *S. nairi*. Subsequently occurrence of *B. nairi* was reported in elephants of India. In addition, in Sri Lanka and other region demonstrated the *S. nairi* (7, 9–12).

There is no comprehensive research on the identification and prevalence rates of intestinal parasites among Iranian zoo animal. This study was designed to determine the parasitic infections among zoo animals based on parasitological and morphometric criteria.

## Materials and Methods

### Study site and animals

This study was conducted in Eram zoological park located in Tehran, Central Iran, where about 120 animal species, including canine, cats, primates, Ruminants, Rodents, Carnivores, Reptiles, Marsupials, Birds and Equine are living. This study was carried out at various families of animals housed in the Eram Park zoo from Oct 2015 to Jun 2015. The study was surveyed on 3 classes of animals (mammals, aves, and reptiles) and 35 different species of animals available in the Eram Park zoo covering Iranian ram, Persian gazelle, Lama, Ostrich, Persian fallow deer (*Cervus dama mesopotamica*), Iguana, Bactrian Camel (*Camelus ferus*), Striped yaena (*Hyaena hyaena*), Persian wild Ass (*Equus hemionus onager*), Jungle cat (*Felis chaus*), Elephant, Wild boar (*Sus scrofa*), Kangaroo, Iranian squirrel, Meerkat, Hedgehog, Red deer (*Cervus elaphus maral*), Olive baboon monkey, Zipi snake, Cobra snake, Los snake, Pythons Albino.

### Sample collection and morphometric studies

One hundred fresh fecal samples were collected from 35 species of animal lived in Eram park zoo in Tehran and were placed into sterile sample bottles. Collection of fecal samples was carried out by the assistance of the animal handlers. Samples were stored at 70% ethanol alcohol and transported to the Medical Parasitological Laboratory of Shahid Beheshti University of Medical Science, Tehran, Iran. Direct wet mount (normal saline and Lugol’s iodine) preparation, formalin-ethyl concentration technique, and permanent stains such as Trichrome and Ziehl Neelsen staining were examined for all suspected samples. The morphometric aspects of isolated eggs were made with the aid of Camera Lucida (×400).

## Results

**Fig. 1:**
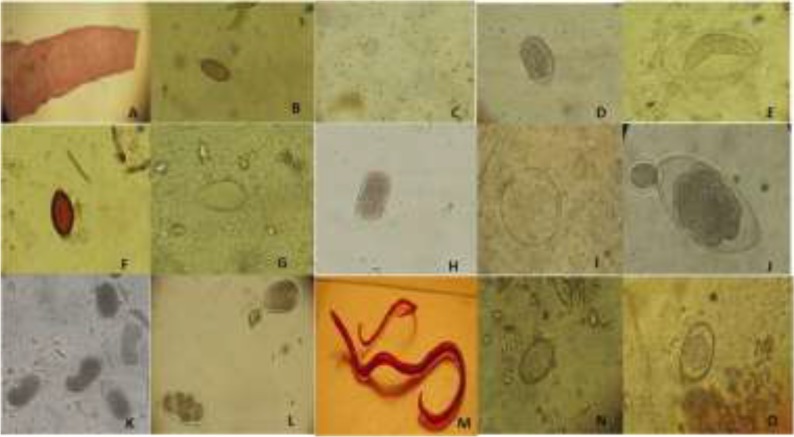
Microscopic observation of intestinal helminthes recovered from animals of Eram Park Zoo. A: Gravid proglottid of *Dipylidium*, B: *Capillaria* egg (Persian fallow deer), C: *Eimeria* oocyst (Iranian ram), D: Trichostrongyloidea egg (Persian wild Ass), E: *Hetrakidae* (iguana), F: *Trichuris* egg (Armenian ram), G: *Strongylous* egg (Ostrich), H: Trichostrongyloidea egg (lama), I: *Dictyocaulus* egg (Humped camel), J:Trichostrongyloidea (Large egg) & *Strongylous* (Small egg) (Persian gazelle), K: Trichostrongyloidea egg (Humped camel), L: *Strongylus* egg, M: *Toxocara* adult worm (jungle cat), N: *Bivitellobilharzia nairi* (elephant) egg, O: *Eimeria* oocyst (Iranian squirrel)

**Fig. 2:**
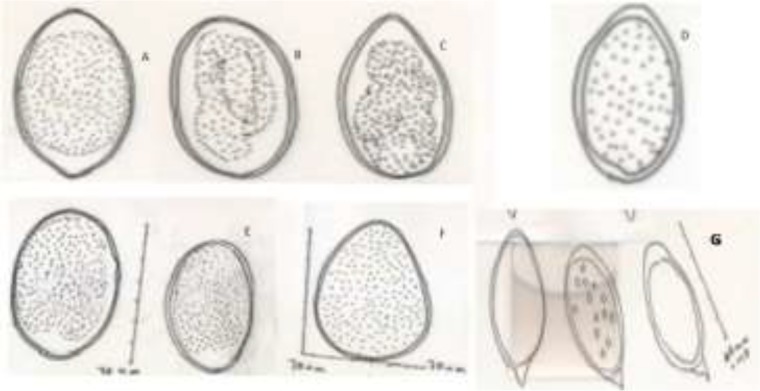
The microscopic and morphometric aspects of drawn intestinal helminths by Camera Lucida. A: *Strongylous* (lama), B: *Strongylus* sp. Egg (Persian gazelle), C: *Strongylous* egg (Bactrian camel (*Camelus ferus*)), D: *Strongylous* egg (Ostrich), E :*Strongylous* egg (Iranian zebra), F: *Strongylous* egg (Ostrich), G*: Bivitellobilharzia nairi* eggs (elephant)

Overall, 23 out of 35 species of zoo animals (Tables 1 and 2) were infected with intestinal parasites (helminths and protozoa) and, whilst no parasitic infection was identified among the Caspian horse, Common Fox, *Vulpes vulpes*, Wolf (*Canis Lupus*), African lion, Bengal Tiger (*Panthera tigris tigris*), *Lynx lynx*, *Ursus arctos*, Golden Eagle, Egyptian vulture, Crowned Crane and Raccoon. 65.7% (23/35) different types of animals were infected with gastrointestinal parasitic. 14 different parasitic infections were observed among the examined animals (Figs. 1 and 2).

**Table 1:** Frequency of intestinal helminthes parasites observed among Zoo animal in Tehran

***No.***	***Animal name***	***Egg/worm***	***Size***
1	Iranian ram	Trichostrongyloidea	110×230μ
2	Iranian ram	*Trichuris* sp.	35×80 μ
3	Persian gazelle	Trichostrongyloidea	110×240 μ
4	Persian gazelle	*Strongylus* sp.	50×100μ
5	Lama	Trichostrongyloidea	150×240μ
6	Lama	*Strongylus* sp.	40×100μ
7	Ostrich	Trichostrongyloidea	70×65μ
8	Ostrich	*Strongylus* sp.	45×68 μ
9	Persian fallow deer (*Cervus dama mesopotamica*)	*Capillaria* sp.	50×25μ
10	Iguana	*Heterakidae* sp.	90×170μ
11	Bactrian Camel (*Camelus ferus*)	Trichostrongyloidea	35×100μ
12	Bactrian Camel (*Camelus ferus*)	*Dictyocaulus viviparus*	85×80μ
13	Striped Hyaena (*Hyaena hyaena*)	*Dipylidium* (gravid proglottid)	-
14	Persian wild Ass (*Equus hemionus onagerr*)	Trichostrongyloidea	50× 70μ
15	Jungle cat (*Felischaus*)	*Toxocara* sp.	Female: 6.9 Cm /Male: 3.5 Cm
16	Elephant (*Elephasmaximus maximus*)	*Bivitellobilharzia nairi*	30×60μ

**Table 2:** Frequency of intestinal protozoan parasites observed in zoo animal in Tehran

***No***	***Animal name***	***Protozoa observed***	***Size***
1	Wild Boar (*Sus scrofa*)	*Blastocystis* sp. & *Iodamoeba* sp.	12–25 μ
2	Kangaroo	*Endolimax nana*	10 μ
3	Kangaroo	*Cryptosporidium* sp.	5μ
4	Iranian ram	*Eimeria* sp.	30×20 μ
5	Iranian squirrel	*Eimeria* sp.	30×20 μ
6	Meerkat	*Blastocystis* sp.	18μ
7	Persian gazelle	*Eimeria* sp.	40×20μ
8	Hedgehog	*Blastocystis* sp.	25μ
9	Red deer (*Cervus elaphus maral*)	*Blastocystis* sp.	32μ
10	Olive baboon monkey	*Entamoeba* cyst	-
11	*Zipi snake*	*Entamoeba* cyst	-
12	Cobra snake	*Blastocystis* sp.	-
13	*Los snake*	*Entamoeba* cyst	-
14	*Pythons Albino*	*Blastocystis* sp.	-

**Fig. 3:**
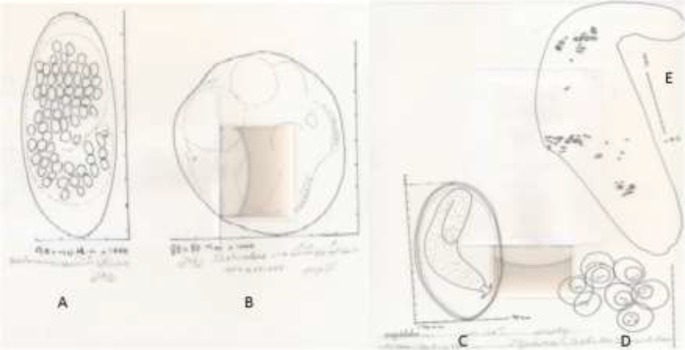
The microscopic and morphometric aspects of drawn intestinal helminths by Camera Lucida. A: *Strongylus* egg (Bactrian camel), B: *Dictyocaulus* egg (Bactrian camel), C: *Hetrakidae* (iguana), D: *Dipylidium caninum* eggs capsule (Hyena), E: *Dipylidium* gravid segment (Hyena

Helminth parasites observed in 13 species of zoo animals (Table 1), while protozoan parasites observed in 12 species (Table 2).Superfamily Trichostrongyloidea (6/16) and *Strongylus* sp. (4/16) were considered as the most prevalent helminth infections as well, *Blastocystis* sp. (6/14), *Entamoeba* cyst (3/14) and *Eimeria* sp. (3/14) were the prevalent protozoan parasites. The microscopic and morphometric aspects of intestinal helminths were drawn by Camera Lucida (Figs. 1–3). Moreover, the oval shapes of *B. nairi* eggs (containing a slightly asymmetrical subterminal spine) with size ranges 60 × 30 μ were identified from an elephant (*Elephasmaximus maximus)* (Fig. 1N and Fig. 2G).

## Discussion

In this study, the various frequency rates of intestinal protozoan and helminthic infections were explicitly isolated and characterized by parasitological and morphometric investigations in Eram park zoo, Tehran where there is no comprehensive study yet.

The majority of the animals studied in this garden were infected with at least one intestinal parasite species. In this study, 65.7% of animal species (mammals, birds, reptiles, amphibians, fish, and etc.) including eight species of helminths (Trichostrongyloidea*, Trichuris* sp., *Strongylus* sp., *Capillaria* sp., *Dictyocaulus*, *Dipylidium* (gravid proglottid), *Toxocara* and *B. nairi*) and six species of protozoan parasites (*Blastocystis* sp., *Iodamoeba, Endolimax nana*, *Cryptosporidium* sp., *Entamoeba* sp. and *Eimeria* sp.) were infected by intestinal parasites. This may be due to suitable conditions of life cycle (temperature, humidity, intermediate hosts, and paratenic hosts., etc.,) of this area, provided the development of intestinal parasites.

In Iran, intestinal parasite observed in working donkeys of North-West of Iran belonged to *Strongylus*, *Parascaris equorum*, *Habronema* sp., *S. edentatus*, *S. equinus* and *S. vulgaris* (13). Animals are infected with various ranges of gastrointestinal parasites, which are representatives of the important pathogenic parasites found in equids worldwide. In the other surveys (Nigeria, Sri Lanka, African Republic, Nepal and Lithuania) some of the intestinal parasites observed in this study have also been reported (1–4, 6–8, 11, 12). Most intestinal parasites observed in these animals can cause intestinal parasitic complications (including; anorexia, vomiting, diarrhea, anemia, loss of weights, etc.,) in humans, particularly animal handlers and visitors (14–18).

In this study, the *B. nairi* egg was detected for the first time from an elephant in Iran. The scanning electron microscope showed the tegument surface of male *B. nairi* is smoother than in other schistosomes. Furthermore, the *B. nairi* egg is similar morphologically to other *Schistosoma* spp, including; *S. hippopotami*, *Ornithobilharzia dattai*, and *O. harinasutai* (12).

Morphologically, the male worms (length: 11.2–13.06 mm) of *B. nairi* are thicker and shorter than female worms (length: 14.1–19.5mm) (12).

There was no significant different among protozoa and helminth prevalence in this garden. In a zoological garden at the Kenya occurrence of helminths (64.4%) reported lower than protozoa (17.1%) and is otherwise with our study (19). In Belgian zoological gardens, the frequency of intestinal nematodes (36.5%) was also identified among captive primates (20).

Among the intestinal parasitic infection, the Trichostrongyloidea and *Strongylus* were the most prevalent helminth infections and *Blastocystis* sp., *Entamoeba* cyst and *Eimeria* sp. were observed in every group of animals studied. All of the parasites observed are zoonosis intestinal parasites and may be gastrointestinal disorders among human (zookeepers and possibility to visitors) and animals.

Zisitors, and zoo officials are exposure to direct contact with the excreta of these animals, therefore, to make sound decisions on regular deworming as well; using improved anthelmintic treatment guidelines should be broadly revised and addressed among the zoo animals and even zookeepers. Employing molecular methods should be employed to identify the accurate species of parasites.

## Conclusion

The high prevalence rate of identified parasitic infections can consider as a economical loss of natural number of zoo animals and threatening source to visitors and workers’ health that have contact with animals or their feces. Therefore, the effective preventive strategies should be considered to establish the risk factors, mechanisms of cross–transmission of parasite, the importance of employing the hygienic practices and well adjusting deworming programs for the animals, zoo workers and visitors.
